# Caffeic Acid Phenethylester Increases Stress Resistance and Enhances Lifespan in *Caenorhabditis elegans* by Modulation of the Insulin-Like DAF-16 Signalling Pathway

**DOI:** 10.1371/journal.pone.0100256

**Published:** 2014-06-25

**Authors:** Susannah Havermann, Yvonni Chovolou, Hans-Ulrich Humpf, Wim Wätjen

**Affiliations:** 1 Institute of Toxicology, Heinrich-Heine-Universität, Düsseldorf, Germany; 2 Institute of Food Chemistry, Westfälische Wilhelms-Universität, Münster, Germany; 3 Institute of Agricultural and Nutritional Sciences, Martin-Luther-Universität Halle-Wittenberg, Halle/Saale, Germany; Rutgers New Jersey Medical School, United States of America

## Abstract

CAPE is an active constituent of propolis which is widely used in traditional medicine. This hydroxycinnamic acid derivate is a known activator of the redox-active Nrf2 signalling pathway in mammalian cells. We used *C. elegans* to investigate the effects of this compound on accumulation of reactive oxygen species and the modulation of the pivotal redox-active pathways SKN-1 and DAF-16 (homologues of Nrf2 and FoxO, respectively) in this model organism; these results were compared to the effects in Hct116 human colon carcinoma cells. CAPE exerts a strong antioxidative effect in *C. elegans*: The increase of reactive oxygen species induced by thermal stress was diminished by about 50%. CAPE caused a nuclear translocation of DAF-16, but not SKN-1. CAPE increased stress resistance of the nematode against thermal stress and finally a prolongation of the median and maximum lifespan by 9 and 17%, respectively. This increase in stress resistance and lifespan was dependent on DAF-16 as shown in experiments using a DAF-16 loss of function mutant strain. Life prolongation was retained under SKN-1 RNAi conditions showing that the effect is SKN-1 independent. The results of CAPE obtained in *C. elegans* differed from the results obtained in Hct116 colon carcinoma cells: CAPE also caused strong antioxidative effects in the mammalian cells, but no activation of the FoxO4 signalling pathway was detectable. Instead, an activation of the Nrf2 signalling pathway was shown by luciferase assay and western blots. CONCLUSION: CAPE activates the insulin-like DAF-16, but not the SKN-1 signalling pathway in *C. elegans* and therefore enhances the stress resistance and lifespan of this organism. Since modulation of the DAF-16 pathway was found to be a pivotal effect of CAPE in *C. elegans*, this has to be taken into account for the investigation of the molecular mechanisms of the traditional use of propolis.

## Introduction

**Figure 1:**
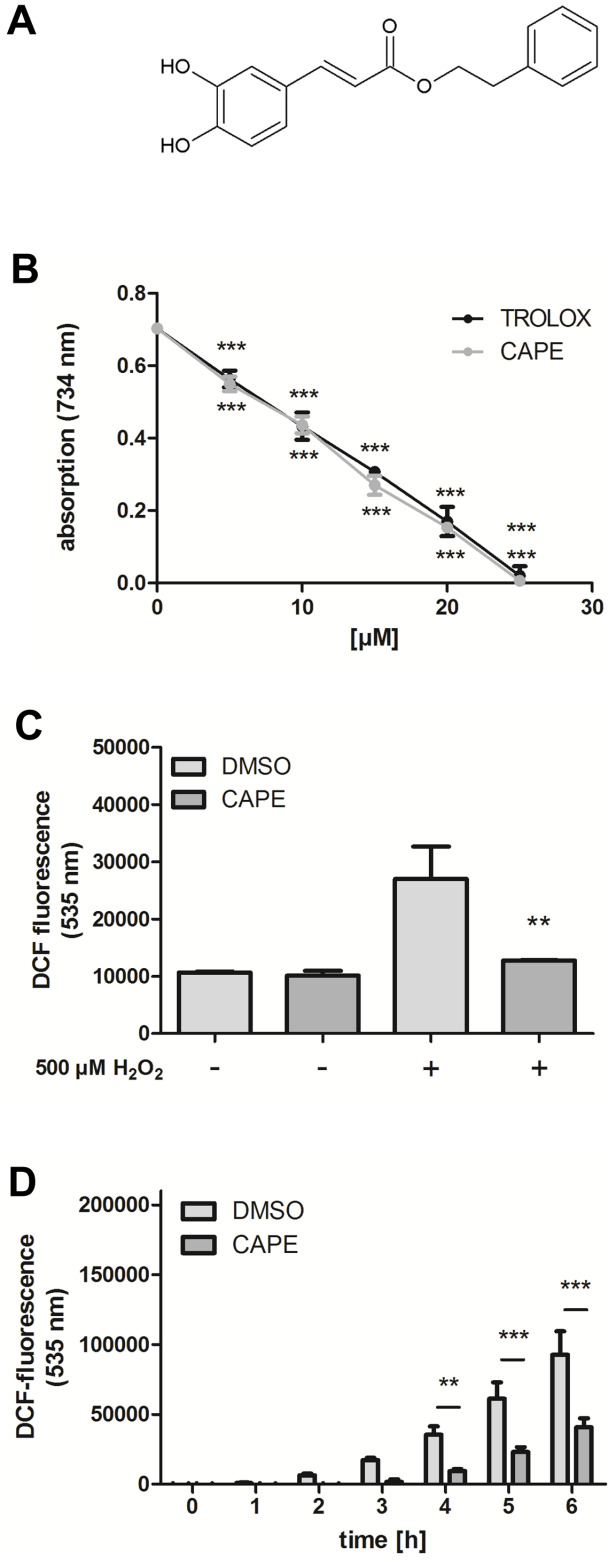
Antioxidant effects of CAPE. (A) Structure of the hydroxycinammic acid derivative. (B) Antioxidant capacity of CAPE in a cell-free system (TEAC): The decrease of the absorption (734 nm) correlates with the ABTS radical scavenging activity; data are the mean ± SD, n = 3. (C) The intracellular ROS accumulation in Hct116 cells was investigated using the fluorescencent probe H_2_DCF-DA. The cells were preincubated with 25 µM CAPE for 4 h, followed by loading with the probe H_2_DCF-DA for 1 h and stress with H_2_O_2_. DCF fluorescence as a marker of intracellular ROS accumulation was measured by flow cytometry; data are the mean ± SD, n = 3, **: p<0.01 versus corresponding DMSO value. (D) The influence of CAPE on ROS accumulation in wild type *C. elegans* was measured using an *in vivo* DCF assay: Nematodes were incubated with CAPE for 2 days and were then subjected to thermal stress (37°C); the DCF fluorescence intensity correlates with the intracellular ROS concentration; data are the mean ± SD, n = 3 with 16 individuals per group and experiment, *: p<0.05 and ***: p<0.001 versus corresponding DMSO-treated group.

The hydroxycinnamic acid derivative caffeic acid phenethylester (CAPE, figure 1A) is a prominent component of propolis (reviewed by [1]). Propolis is a natural product which honey bees (*apis mellifera*) produce from plant resins. The exact chemical composition of propolis varies greatly depending on the phytogeographical areas and the season. In traditional and folk medicine propolis is widely used for the treatment of different diseases, due to a broad spectrum of pharmacological properties, e. g. antimicrobial, immunomodulatory, anticancer, hepatoprotective, neuroprotective and anti-inflammatory activities (reviewed by [2]–[4]). For this reason, propolis is recognised to have the potential for the development of new drugs (reviewed by [5]). CAPE, one of the major constituents exhibits beneficial effects too: It was shown that this compound possesses cytotoxic and pro-apoptotic activities [6] and that these cytotoxic effects were preferentially mediated in tumour cells [7]. Moreover, [8] demonstrated that CAPE exerts an anti-tumour effect *in vivo*: This compound was able to inhibit the TPA-induced tumour promotion in mouse skin. Further anticancer effects of propolis and CAPE are reviewed by [9] and [10], respectively. At the molecular level CAPE has been shown to be a potent and specific inhibitor of the proinflammatory NFκB signalling pathway [11]. Next to direct antioxidant properties [12] CAPE is also known to activate the redox-sensitive Nrf2 pathway in mammalian cells, as shown e.g. renal epithelial cells [13] and astrocytes [14] and in a rat model *in vivo* [15].

Propolis is traditionally used to treat diverse diseases, e. g. premature ageing [16] and also for CAPE, protecting effects against aging-related damage in Sprague Dawley rats were demonstrated [17], [18]. Since the molecular mechanisms of these “anti-ageing” effects are largely unknown, we used the nematode *Caenorhabditis elegans* to investigate the effects of the active component CAPE on oxidative stress, stress resistance and lifespan. The model system *C. elegans* is getting increasingly important in science as a relatively simple model system to analyse effects of natural compounds *in vivo*. We have focused on the influence of CAPE on the modulation of two central redox-active signalling pathways in the nematode.

A variety of detoxifying enzymes is up-regulated after exposure to oxidative stress. The major redox-sensitive transcription factor regulating such enzymes in mammals is Nrf2 [19]. The functionality of Nrf2 is conserved across species and is known e.g. in chicken, mouse, rats, humans and also in model organisms like *D. melanogaster* and *C. elegans* [20]. Though sequence homology between human Nrf2 and its *C. elegans* counterpart SKN-1 is relatively low and mechanistic differences are known, both bind to antioxidant responsive elements after activation by oxidative stress and therefore modulate target gene expression [21]. Positive effects like an increased stress tolerance [22] and life prolongation [23] are connected with SKN-1 activity. It has already been shown that distinct polyphenolic Nrf2 activators are able to modulate SKN-1 activity [24].

Another important stress responsive transcription factor in *C. elegans* is DAF-16. The activity of this longevity mediating transcription factor is mainly regulated by the insulin-like signalling pathway. DAF-16 is the *C. elegans* orthologue to the mammalian FoxO proteins [25]. Amongst others it controls the expression of a set of important antioxidative enzymes like, e. g. manganese superoxide dismutase SOD-3 and the catalases CTL-1 and CTL-2 [26], [27] and has previously been shown to respond to certain natural compounds [28].

We have analysed the effects of CAPE on the activity of SKN-1 and DAF-16, as well as the modulation of reactive oxygen species (ROS) accumulation, stress resistance and lifespan in *C. elegans*. Furthermore, the molecular mechanisms found in the multicellular organism C. elegans were compared with molecular effects detected in a human cell culture model (Hct116 cells).

## Material and Methods

### Material

*C. elegans* strains used in this study are N2 wild type, transgenic *C. elegans* LD001 (Pskn-1::SKN-1::gfp; rol6) and CF1038 (daf-16(mu86) I.). Some of these strains and OP50 and streptomycin resistant OP50-1 *Escherichia coli* strains were provided by the *Caenorhabditis* Genetics Centre, which is funded by NIH Office of Research Infrastructure Programs (P40 OD010440). Nematodes were held at 20°C on nematode growth medium (NGM) plates with OP50 as a food source as described by [29]. HT115 *Escherichia coli* for RNAi experiments were kindly provided by Prof. Olaf Bossinger (University of Aachen).

Hct116 human colon carcinoma cells were obtained from the DSMZ (Braunschweig, Germany). Cells were cultured at 37°C in a humidified atmosphere of 5% CO_2_ using high glucose DMEM supplemented with 10% fetal calf serum, 100 U/ml penicillin and 100 µg/ml streptomycin.

The luciferase reportergene vector (ARE GST-Ya) was kindly provided by Dr. Ming Zhu (UC Davis Cancer Center, California, USA) and was constructed as described elsewhere [30].

### Antioxidative effects

#### a) TEAC-assay

A radical solution was prepared using same volumes of a 14 mM ABTS solution and a 4.9 mM APS solution which was diluted with 80% ethanol until it had an absorption of 1.4 at 734 nm. The trolox calibration and different concentrations of CAPE were prepared in 80% ethanol. Sample and radical solution were mixed and the absorption was measured spectrophotometrically (734 nm) after 2 min.

#### b) *In vivo* DCF assay (*C. elegans*)

Synchronisation of the culture was achieved by placing gravid adults on NGM plates and allowing them to lay eggs for up to 3 h. After this time the adults were removed and the eggs were allowed to hatch and develop to larval stadium L4 or young adult before incubation with 100 µM CAPE (100 mM stock in DMSO) or an equivalent volume of DMSO, respectively. Incubation was performed at 20°C in liquid NGM containing 1% BSA, 50 µg/ml Streptomycin and 1×10^9^ OP50-1/ml as a food source. After 2 days treatment with daily changes of incubation medium the nematodes were individually transferred into the wells of a 384-well plate already containing M9 buffer. H_2_DCF-DA was added to a final concentration of 50 µM and the plate was sealed against evaporation. 37°C thermal stress was applied and fluorescence at 535 nm (excitation wavelength 485 nm) was measured once an hour using a plate reader (Wallace Victor^2^ 1420 multilabel counter, Perkin-Elmer, Wellesley, USA).

#### c) *In vitro* DCF assay (cell culture)

Cells were seeded into 6-well plates (5×10^5^ cells/well) and were allowed to attach for 24 h. Cells were treated with 25 µM CAPE or DMSO as vehicle control for 4 h, were washed with medium and were then loaded with 10 µM 2,7-dichlorodihydrofluorescein diacetate (H_2_DCF-DA; Sigma Deisenhofen, Germany) for 15 min. After washing with medium the cells were stressed for 1 h with 500 µM H_2_O_2_ and were then harvested for flow cytometry using an accuri C6 flow cytometer (BD Biosciences). Exitation was 488 nm, emmision was measured at 530±15 nm.

#### d) Catalase activity assay

To determine the catalase activity, the decomposition of H_2_O_2_ was spectrophotometrically measured at 25°C. Homogenized nematodes (Minilys Tissue Homogenizer, Bertin Technologies) were added to an incubation mix (phosphate buffer 50 mM, pH 7.0 and 1% triton X-100) to solubilize membranes. H_2_O_2_ (25 mM) was added and the decrease of absorbance (240 nm) was analysed. The specific catalase activity was calculated (molar extinction coefficient 43.6 for H_2_O_2_) and expressed as units (decomposition of 1 µmol H_2_O_2_ per minute) per mg protein.

### Modulation of redox-sensitive signalling pathways

#### a) Detection of nuclear translocation by fluorescence microscopy (DAF-16, SKN-1)

Transgenic *C. elegans* LD001 or CF1038 were held at 20°C, synchronised as described above and transferred into liquid NGM containing 1% BSA, 50 µg/ml Streptomycin and 1×10^9^ OP50-1/ml at day 3. They were incubated for 1 h with 100 µM CAPE or an equivalent volume of DMSO and were then mounted onto microscope slides, anaesthetised using 10 mM sodium azide and analysed swiftly. 30 Nematodes were counted concerning visibility of GFP-fluorescent nuclei in the intestinal cells for SKN-1 localisation or visibility of nuclei all over the body for DAF-16 localisation.

#### b) Detection of nuclear translocation by western blot and immunostaining (Nrf2, FoxO)

Hct116 cells were seeded into 6-well plates (7.5×10^5^ cells/well) and were allowed to attach for 24 h. Proteins were isolated after treatment with different concentrations of CAPE for 4 h. For total protein cells were washed with ice-cold PBS and suspended in ice-cold RIPA buffer. After two freeze-thaw-cycles lysates were centrifuged and the supernatant containing the proteins was removed. For nuclear and cytosolic fraction cells were washed with ice-cold PBS and lysed for 15 min in ice-cold buffer A (10 mM HEPES, 10 mM KCl, 0.1 mM EDTA, 0.1 mM EGTA, 1 mM DTT, 0.5 mM PMSF, Proteinase Inhibitor Cocktail and 0.01% okadaic acid) before adding Nonidet-P40 and vortexing for 1 min. After pelleting the lysate by centrifugation the supernatant containing the cytosolic fraction was removed. The pellet was rinsed in ice-cold PBS and was then stirred in buffer B (20 mM HEPES, 400 mM KCl, 1 mM EDTA, 1 mM EGTA, 1 mM DTT and 1 mM PMSF) at 4°C for 25 min before centrifugation. The supernatant contained the nuclear fraction. Protein concentrations were measured using the Bio-Rad DC protein assay following manufactureŕs instructions. 40 µg total protein, 30 µg nuclear or 60 µg of cytosolic fraction for detection of Nrf2 and 50 µg total protein or 60 µg of protein fractions for detection of FoxO4 were separated by SDS-PAGE and were then transferred to PVDF western blot membranes (Roche, Mannheim, Germany). Membranes were blocked in 5% BSA in TBS supplemented with 0.1% Tween 20 (TBST) for 1 h at room temperature and were then probed with Anti-Nrf2 (1∶5000, Epitomics, Burlingame, CA) or Anti-FoxO4 (1∶1000, Cell Signaling) antibodies, respectively. The secondary antibody was Goat Anti-Rabbit (1∶3000, SouthernBiotech, Birmingham, AL). Signals were visualised using BM Chemiluminescence Western Blotting Kit (Roche, Mannheim, Germany) and detected with a Fusion FX7 imaging system (Peqlab, Erlangen, Germany). Densitometric analysis was performed using the Bio1D software by Peqlab (Erlangen, Germany).

#### c) Analysis of ARE activation (Nrf2, reporter gene assay)

Transient transfection was performed with a batch protocol using JetPei™ transfection reagent (Polyplus Transfection) based on the manufacturer's protocol. In brief, complex forming was carried out with 2 µl of transfection reagent per µg plasmid DNA. The complexes were mixed with 5×105 Hct116 cells and were seeded into 6- well plates. After 24 h of transfection cells were incubated with different concentrations of CAPE for another 24 h. Cells were rinsed with ice-cold PBS and were then lysed by shaking 15 minutes in Reporter Lysis Buffer (Promega) before centrifugation. Luciferase activity in the supernatant was tested using the Luciferase Assay Kit (BioThema AB) according to the manufacturer's protocol in a fluorescence reader equipped with a dispenser (Wallace Victor2 1420, Perkin-Elmer, Wellesley, USA). The chemiluminescence was normalised by the protein content measured using the Bio-Rad Protein Assay (Bio-Rad).

### Stress resistance (SYTOX Assay)

*C. elegans* were synchronised by egg laying and treated with 100 µM CAPE or DMSO for 2 days starting from day 3 as described above for the *in vivo* DCF assay. Afterwards the nematodes were washed with PBS containing 0.1% Tween 20 and were individually transferred into the wells of a 384-well plate already containing PBS. SYTOX Green nucleic acid stain was added to a final concentration of 1 µM and the plate was sealed against evaporation. 37°C thermal stress was applied and fluorescence at 535 nm (excitation wavelength 485 nm) was measured every 15 minutes using a fluorescence reader (Wallace Victor^2^ 1420, Perkin-Elmer, Wellesley, USA). The increase of fluorescence intensity was used to calculate so called “virtual time points of death”: An individual was considered dead if its fluorescence intensity was more than 3 times higher than the mean of its initial 3 fluorescence values.

### Lifespan analysis

N2 wild type *C. elegans* were synchronised by egg laying and were treated with 100 µM CAPE or DMSO as described above at day 3. Survival of the nematodes at 25°C was examined daily by response to touching first posterior and then anterior. Non-responding nematodes were further cut in two. *C. elegans* that were scored as alive were transferred daily into fresh incubation medium during the time of progeny and further on every second day at the minimum. Nematodes displaying internal hatching or extruded internal organs were excluded from the analysis. For lifespan analysis under RNAi conditions N2 or CF1038 nematodes were synchronised as described above but using HT115 empty vector or HT115 SKN-1 RNAi bacteria as a food source on agar plates containing 12.5 µg/ml tetracycline, 100 µg/ml ampicillin and 1 mM Isopropyl-β-D-thiogalactopyranosid (IPTG; Promega, Madison, Wisconsin, USA). The liquid NGM contained 1% BSA, 12.5 µg/ml tetracycline, 100 µg/ml ampicillin, 1 mM IPTG and 1×10^9^ respective HT115/ml.

### Statistical analysis

Results are expressed as mean ± standard deviation. All statistical analysis was performed using GraphPad Prism 5 software (La Jolla, USA). The minimum level of significance was p<0.05. Statistical significance was assessed by unpaired Students *t* test with two-sided testing. Where appropriate, instead One-way ANOVA with Dunnet's post-test or Two-way ANOVA with Bonferroni post-tests were used. Lifespan analysis was performed using Kaplan Meier statistics. Nematodes that were lost, showed internal hatching or were killed during mechanical viability testing were discriminated.

## Results

### Antioxidative capacity of CAPE

We first estimated the antioxidative capacity of CAPE in a simple cell free system (TEAC assay: trolox equivalent antioxidative capacity assay). In this assay, the ability of a compound to decolourise a green radical solution is compared to that of the synthetic antioxidant trolox. As shown in fig. 1B, CAPE possesses a significant radical scavenging potential already at a concentration of 5 µM. The compounds radical scavenging ability of CAPE was comparable to that of the synthetic vitamin E derivative which was used as a positive control. Next we analysed if CAPE was also capable of decreasing ROS in a cellular system (Hct116 colon carcinoma cells) useing the ROS-sensitive probe DCF. After oxidation, this probe shows a bright fluorescence which was used as marker of intracellular ROS concentration. Incubation of Hct116 cells with CAPE (25 µM, 4 h) did not have any effect on DCF fluorescence, whereas incubation with H_2_O_2_ (500 µM, 1 h) resulted in a strong increase in DCF fluorescence (fig. 1C). A preincubation with CAPE (25 µM, 4 h) strongly prevented the H_2_O_2_-mediated increase in DCF fluorescence almost to basal levels. Next we investigated if this compound is also able to reduce intracellular ROS concentrations in the *in vivo* model system *C. elegans*. The nematodes were pretreated with CAPE (100 µM) for two days, then the worms were placed individually into the wells of a 384-multiwell plate before adding the ROS-sensitive probe H_2_DCF-DA and placing the worms in a fluorescence reader at 37°C. This treatment results in a strong fluorescence increase in the DMSO control group. CAPE decreased this ROS-mediated fluorescence increase dose-dependently (supplementary file). To the best of our knowledge this is the first work that showed that pretreatment of *C. elegans* with CAPE strongly lowered the DCF fluorescence: In comparison to the DMSO control group the DCF fluorescence after CAPE treatment was in the order of less than 50% of the control value (fig. 1D). To investigate the effect of CAPE on antioxidant enzymes, we analysed the activity of the central antioxidative enzyme catalase. However, treatment with CAPE (100 µM, 48 h) did not change the activity of this enzyme: The catalase activity in wild-type nematodes treated with DMSO was determined as 2.068+/−0.29 U/min/mg protein, while the activity of CAPE-treated nematodes was 1.962+/−0.24 U/min/mg protein (no significant difference).

### Influence of CAPE on SKN-1/Nrf2 signalling

**Figure 2:**
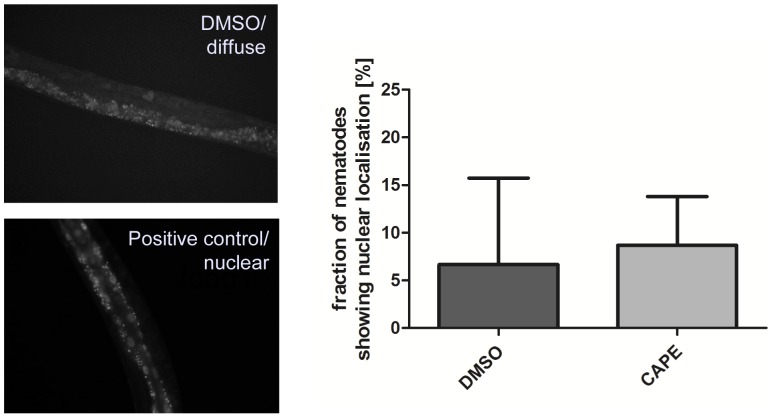
CAPE does not induce translocation of SKN-1::GFP into nuclei of intestinal cells in *C. elegans*. 3-day-old synchronised LD001 *C. elegans* were treated with vehicle control or 100 µM CAPE (or baicalein as positive control) for 1 h at 20°C and were then analysed by fluorescence microscopy concerning visibility of GFP-fluorescence in nuclei of the intestinal cells as observed. The fraction of nematodes showing nuclear GFP localisation was determined (DMSO: 3%/0%/17%; CAPE: 10%/3%/13%); mean ± SD, 30 individuals per group in each of the three independent experiments.

**Figure 3:**
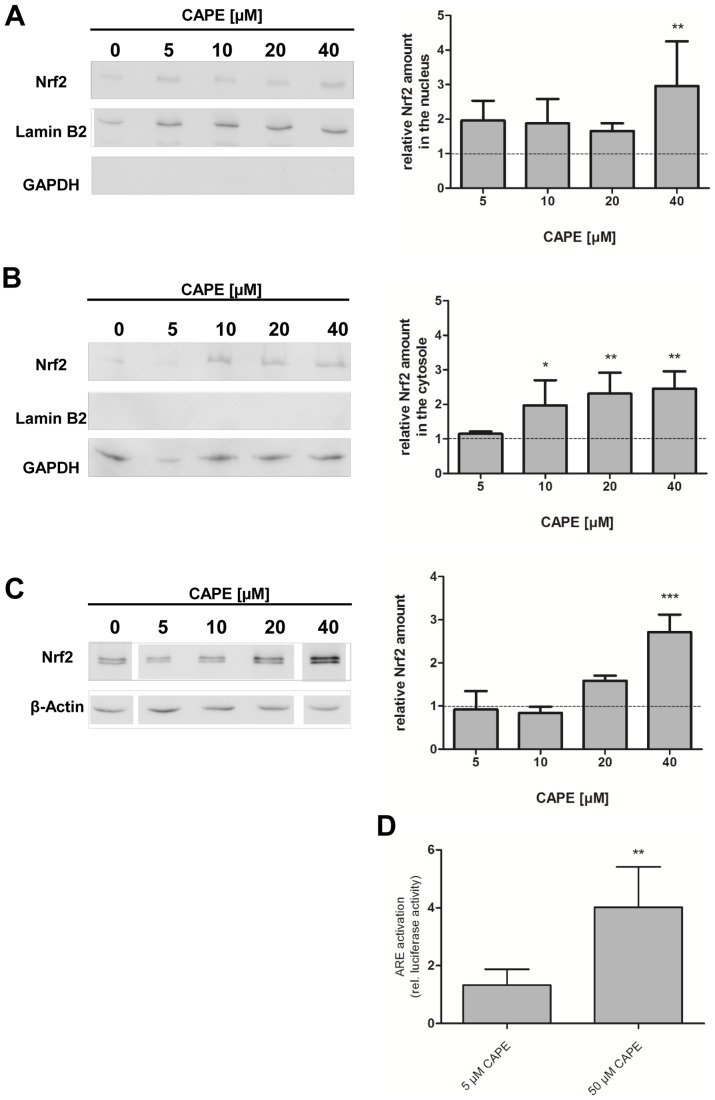
CAPE activates the Nrf2 pathway in Hct116 cells. Cells were incubated with different concentrations of CAPE for 4(A) nuclear and (B) cytosolic protein fractions or (C) total protein. Antibodies against Lamin B2 (nuclear marker) and GAPDH (cytosolic marker) were used as control for the quality of the fractionation process, while β-Actin was used as a loading control. One representative blot of three is shown, data (mean ± SD) are given as fold increase of Nrf2 protein amount compared to the vehicle control, *: p<0.05, **: p<0.01 and ***: p<0.001. (D) Cells were transfected with an ARE luciferase construct and then incubated with different concentrations of CAPE for 24 h. Luciferase activity is shown, data are the mean ± SD, n = 3, **: p<0.01 versus DMSO-treated control.

Polyphenolic compounds can reduce intracellular ROS levels i) by direct antioxidative ( = radical scavenging) mechanisms and/or ii) indirectly by induction of antioxidative enzymes. The later resulting in a more effective antioxidative defence system for the cell or organism. To investigate if CAPE also exerts indirect antioxidative effects in *C. elegans*, we analysed the effect of this compound on the intracellular localisation of the Nrf2-homologue SKN-1 in the intestinal cells of the nematode using a transgenic strain expressing SKN-1::GFP. An activation of this redox-active signalling pathway requires a translocation of SKN-1 into the nucleus. The transcription factor was classified as inactive if the nematodes showed a diffuse fluorescence and active if it displayed a fluorescence localised in the nuclei. As depicted in fig. 2, the localisation of SKN-1 during basal conditions (DMSO-treated nematodes) mainly show a diffuse GFP localisation (only 7% of the nematodes with nuclear localisation detectable). Treatment with a positive control (shown here is incubation with 100 µM Baicalein, see [24]) leads to an increase in the nuclear SKN-1::GFP localisation. Incubation with 100 µM CAPE for 1 h did not influence the fraction of nematodes showing nuclear SKN-1 localisation compared to the DMSO-treated control: The amount of worms showing a nuclear localisation of the transcription factor was about 9%. Since it has been reported that CAPE is an activator of the Nrf2-ARE signalling pathway in mammalian cells [13], [14], we proofed this by additional experiments using Hct116 human colon carcinoma cells: By analysis of the localisation of this transcription factor (western blot analysis of nuclear and cytosolic fractions), a concentration-dependent accumulation of Nrf2 was detected both in the nuclear and cytosolic fraction (fig. 3A and 3B) and consistently, also in the total protein content (fig. 3C). This increased concentration of Nrf2 in the nucleus is associated with an increased transcriptional activity: Incubation with 50 µM CAPE for 24 h leads to an approximately four-fold increase in luciferase activity using a ARE luciferase assay (fig. 3D). To investigate if the CAPE-mediated Nrf2 activation is exclusively detectable in carcinoma cells we also performed an analysis using FHC immortalised normal fetal colon cells. In this case, an incubation with CAPE (50 µM, 4 h) also leads to a significant increase in the amount of Nrf2 (data not shown).

### Influence of CAPE on DAF-16/FoxO signalling

**Figure 4:**
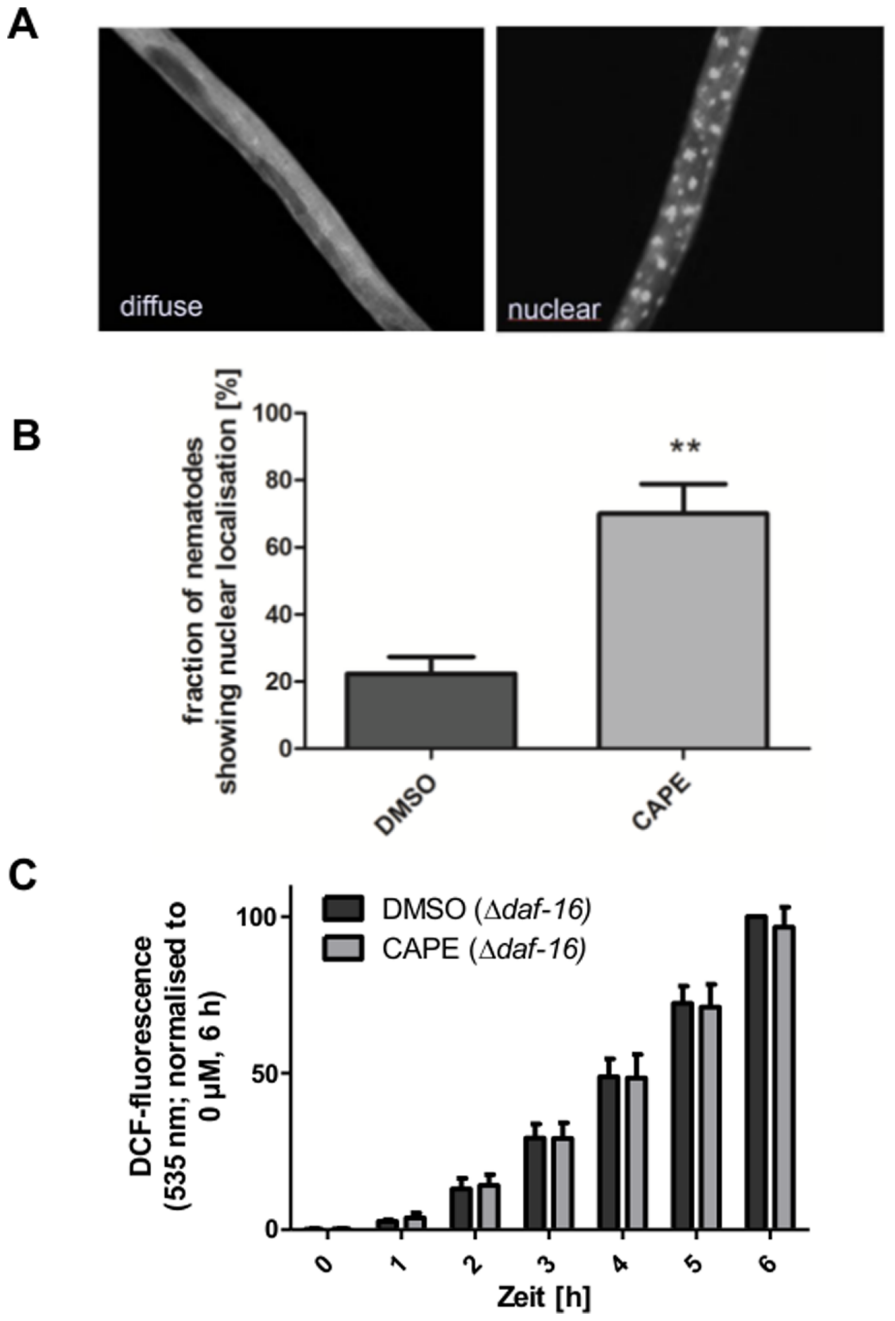
CAPE induces translocation of DAF-16::GFP in *C. elegans* which is responsible for reduction of oxidative stress. 3-day-old synchronised TJ356 *C. elegans* were treated with vehicle control or 100 µM CAPE for 1 h at 20°C and were then analysed by fluorescence microscopy concerning visibility of GFP-fluorescence in nuclei (A). B: The fraction of nematodes showing nuclear GFP localisation was determined; mean ± SD, 30 individuals per group in each of the three independent experiments, **: p<0.01, C: The influence of DAF-16 on CAPE-mediated reduction of ROS accumulation was measured using the transgenic DAF-16-mutant strain CF1038 (*in vivo* DCF assay): Nematodes were incubated with CAPE for 2 days and were then subjected to thermal stress (37°C); the DCF fluorescence intensity correlates with the intracellular ROS concentration; data are the mean ± SD, n = 3 with 16 individuals per group and experiment, *: p<0.05 versus corresponding DMSO-treated group.

Since the SKN-1 signalling pathway was not modulated by CAPE in *C. elegans*, we analysed if this compound may modulate DAF-16, another pivotal redox-sensitive transcription factor in *C. elegans*. This transcription factor which is a component of the insulin-like signalling pathway is also connected with the control of oxidative stress, longevity and ageing. To analyse the effect of CAPE on the DAF-16-localisation, we used a transgenic strain (TJ356) which expresses a DAF-16::GFP construct. Activation of this transcription factor also requires a translocation in the nucleus which can be analysed similar to the SKN-1-activation by fluorescence microscopy. CAPE (100 µM, 1 h) significantly increased the fraction of worms exhibiting nuclear accumulation of DAF-16 from 22% (DMSO-treated control) to 70% (fig. 4).

**Figure 5:**
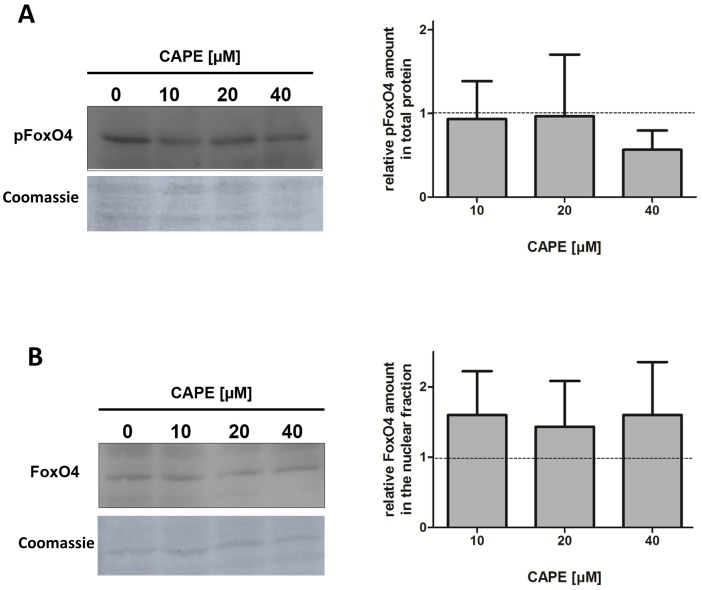
CAPE does not activate FoxO4 in Hct116 cells. Cells were incubated with different concentrations of CAPE for 4(A) total protein or (B) protein fractions. The amount of phosphorylated FoxO4 (pSer194) in total protein and the amount of total FoxO4 in the nuclear fraction were determined. One representative out of three is shown, data (mean ± SD) are given as fold increase of FoxO4 protein amount compared to the vehicle control.

Since in the case of SKN-1/Nrf2 a discrepancy in the molecular mechanisms of mammalian cells and the nematode was detected, we performed an additional experiment analysing the effect of CAPE in Hct116 cells. The activity of mammalian FoxO proteins (orthologues of DAF-16) is regulated by inhibitory phosphorylation which leads to a nuclear export. We examined the effect of CAPE on the activation of the isoform FoxO4 (western blot). First, we analysed the effect of CAPE (up to 40 µM) on the amount of Ser194-phosphorylated FoxO4. As shown in fig. 5A, incubation with CAPE has no influence on phosphorylation at this position, only a slight tendency could be seen at the highest concentration used. We further analysed the effects on the localisation of the transcription factor using western blot of the nuclear and cytosolic protein fractions (fig. 5B). While no FoxO4 was detectable in the cytosol the nuclear fraction showed a constant FoxO4 concentration. Under these experimental conditions no translocation and therefore no activation was detectable.

### CAPE increases thermotolerance in *C. elegans*

**Figure 6:**
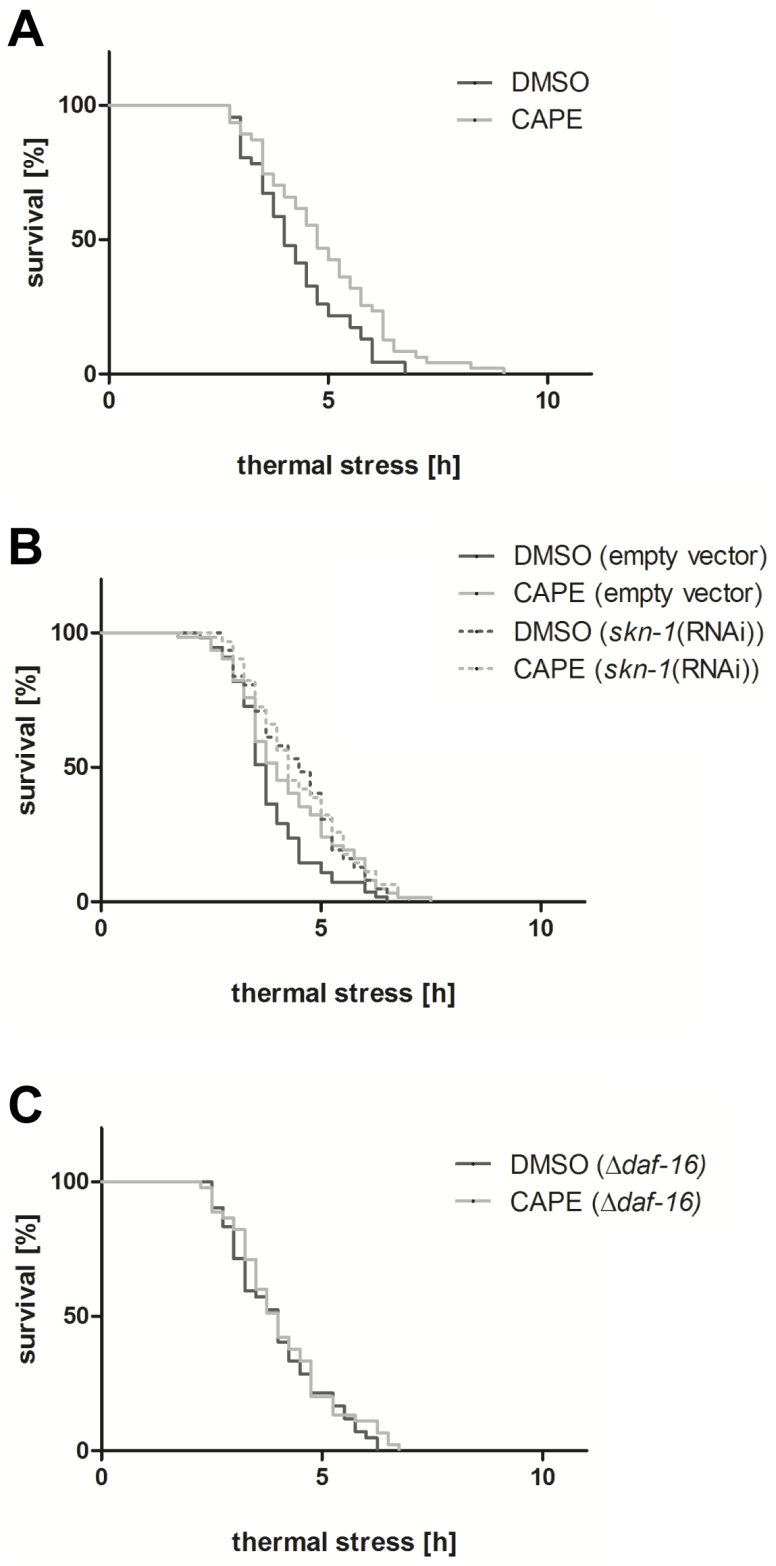
CAPE enhances *C. elegans* resistance to thermal stress in dependence on SKN-1 and DAF-16. (A) Wildtype C. elegans (B) wildtype C. elegans under SKN-1 RNAi conditions and (C) CF1038 DAF-16-mutant C. elegans were incubated with CAPE for 2 days and were then subjected to thermal stress (37°C); the increase of the SYTOX Green fluorescence intensity was used to define “virtual time points of death”; Kaplan-Meier statistics were performed with a total of 48 nematodes per group in three independent experiments.

Activation of certain protective signalling pathways is suggested to increase the stress-resistance of organisms (e.g. thermotolerance). Therefore, we analysed the influence of CAPE on the survival of *C. elegans* which were exposed to lethal thermal stress (37°C) using the fluorescent probe SYTOX Green. This membrane-impermeable dye intercalates in the DNA leading to a strong increase in fluorescence. Since the dye cannot diffuse across intact cellular membranes, an increase in fluorescence is only possible if cell membrane integrity is lost which was used as a marker to distinguish alive and dead nematodes. Preincubation of the nematodes with 100 µM CAPE significantly increases their thermotolerance as shown in fig. 6A. To further elucidate if stress-sensitive signalling networks are required for this effect we repeated the assay using either the mutant strain CF1038 which does not express functional DAF-16 or wildtype nematodes fed on SKN-1 RNAi-bacteria. The loss of SKN-1 due to RNAi inhibited a prolonged thermotolerance induced by incubation with 100 µM CAPE (fig. 6B). Further, in the DAF-16 mutant *C. elegans* pretreatment with 100 µM CAPE also did not lead to an increased thermotolerance anymore (fig. 6C).

### CAPE prolongs *C. elegans* lifespan

**Figure 7:**
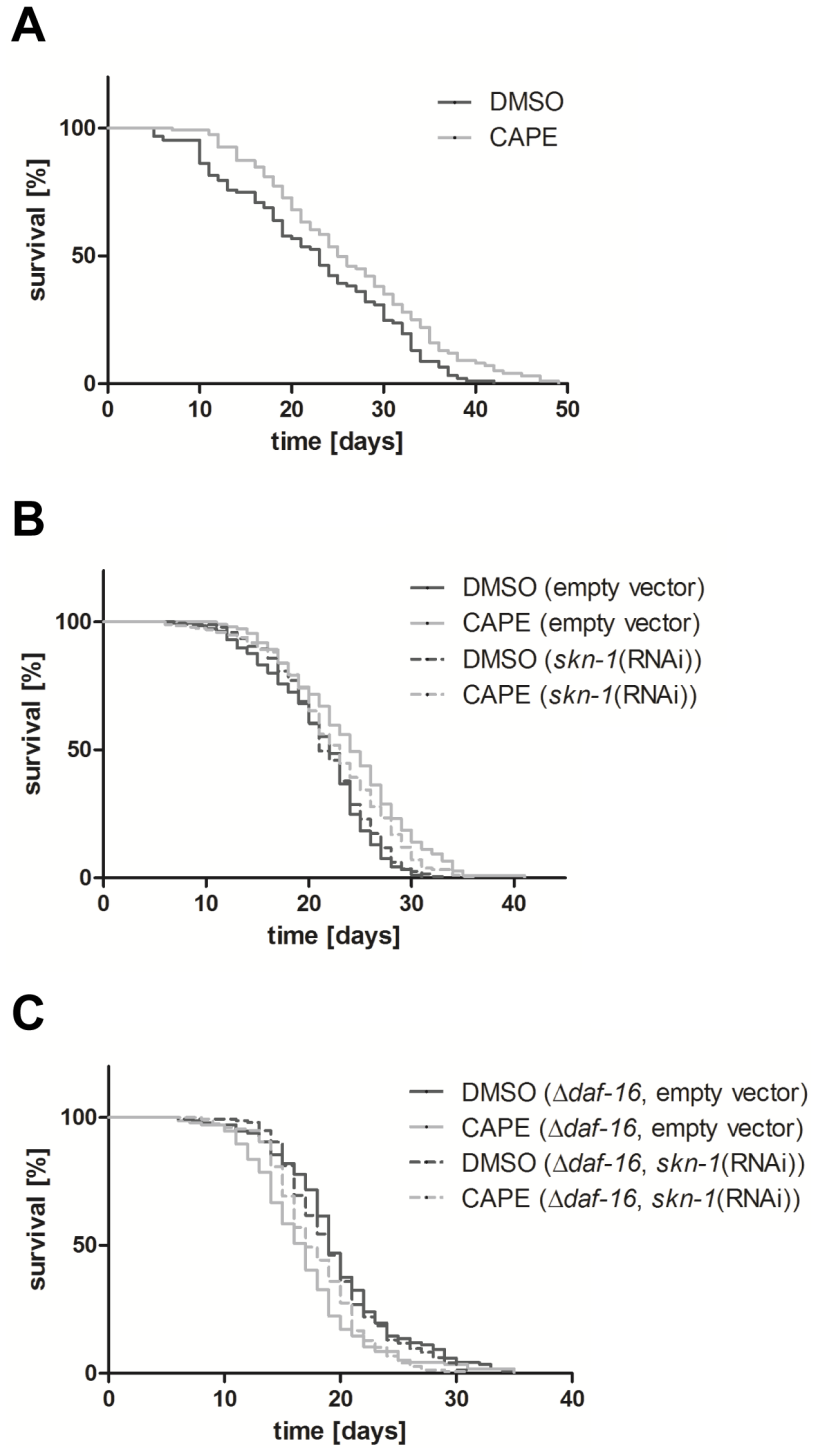
CAPE prolongs the lifespan of C. elegans. (A) Wildtype C. elegans, (B) wildtype C. elegans under SKN-1 RNAi conditions and (C) CF1038 DAF-16-mutant *C. elegans* under SKN-1 RNAi conditions were treated with 100 µM CAPE or vehicle. The nematodes were kept at 25°C and survival was determined by touch-provoked movement. Kaplan-Meier statistics were performed with a total of 120–200 individuals in 3–5 independent experiments.

Since enhanced stress resistance in many cases is also associated with an improvement of lifespan, we performed a lifespan assay with *C. elegans* incubated with 100 µM CAPE (fig. 7A). Incubation of *C. elegans* with this hydroxycinnamic acid derivative resulted in a significant prolongation of the median and maximum lifespan by 9% and 17%, respectively compared to the vehicle-treated control group. To identify the molecular mechanism of this effect, we performed the lifespan analysis under SKN-1 RNAi-conditions (fig. 7B). Since the lifespan of the nematodes was significantly increased by CAPE in this group too, it has to be suggested that CAPE-mediated life prolongation is independent of the functionality of SKN-1. We further investigated the longevity effect of CAPE using a DAF-16 loss of function mutant *C. elegans* strain (CF1038). In this strain, an incubation with CAPE led to a significant reduction of the median lifespan by 11% compared to the corresponding control showing a correlation between functionality of DAF-16 and prolongation of lifespan by CAPE. In a third experiment, we analysed the effect of a combination of SKN-1 knock-down (RNAi) and DAF-16 loss of function on CAPE-mediated lifespan prolongation (fig. 7C). However, the additional loss of SKN-1 did not have any further influence compared to the effect of the DAF-16 loss of function transgenic strain alone.

## Discussion

Distinct natural compounds have important physiological effects on organisms and especially on the intestinal cells as the site of uptake. For these cells the interaction of food compounds with the intracellular signalling network is of critical importance. We have therefore analysed the antioxidative effects of CAPE, a constituent of propolis which is used in traditional medicine and its impact on the model organism *C. elegans*. Our main focus of interest next to positive effects on ROS level, thermotolerance and lifespan was set on the molecular mechanisms that mediate these effects. The idea that protective effects are not caused by direct antioxidative effects but modulation of transcription factor activity is currently widely accepted [31], [32]. CAPE shows a direct antioxidative potential similar to a synthetic vitamin E derivative, the reference compound used. This agrees with results published by [12] who could also show an antioxidant capacity in several cell free systems. To the best of our knowledge we were the first investigating antioxidative effects of CAPE in *C. elegans*: The compound showed no antioxidative effect under basal conditions, but significantly reduced the accumulation of ROS under stress. However, the antioxidant effect of CAPE was not due to an increased activity of the antioxidative enzyme catalase. A similar effect was detectable in mammalian cells: In Hct116 cells, CAPE inhibited the accumulation of ROS induced by H_2_O_2_. A comparable effect of CAPE has previously been shown in human hepatoma cells after incubation with tert-butylhydroperoxide [33]. In addition to reduced ROS accumulation detected after incubation with CAPE, we were able to show that the nematode's resistance to thermal stress was significantly increased. Further, we were able to demonstrate that *C. elegans* shows a significantly longer lifespan: median lifespan was prolonged from 23 to 25 days and the maximum lifespan from 42 to 49 days. Since life prolongation is usually attributed to activation of certain signalling pathways and not to pure antioxidant activity we analysed the impact of CAPE on the activation of two central ageing related transcription factors SKN-1 and DAF-16. An incubation of a SKN-1::GFP expressing transgenic strain with CAPE did not lead to a nuclear localisation of the transcription factor showing that CAPE does not interact with this pathway. Although CAPE did not induce nuclear translocation of SKN-1::GFP in *C. elegans*, enhanced resistance to thermal stress by CAPE is dependent on SKN-1. This finding may be due to adaptive mechanisms or a generally low stress resistance in the SKN-1 knockdown nematodes which cannot further be modulated by CAPE. We further performed a lifespan analysis under SKN-1 RNAi conditions. The knock-down of SKN-1 did not repress the CAPE mediated life prolongation therefore showing that the effects of CAPE on *C. elegans* lifespan are SKN-1 independent. This result was unexpected since activation of the SKN-1 homologue Nrf2 by CAPE has been published previously [34]. Therefore we also investigated the influence of CAPE on the Nrf2-pathway using Hct116 human colon carcinoma cells. Western blot of total protein isolated from CAPE treated cells showed a concentration dependent increase of Nrf2 indicating a modulation. In parallel, the Nrf2-level also increased in the cytosolic and nuclear fractions. Further we analysed transcriptional activation using an ARE-luciferase reporter gen assay. It could be demonstrated that an incubation with CAPE results in a 4-fold higher reporter gene expression compared to control cells. This result is in accordance to the study by [34]. Taken together, it could clearly be shown that CAPE is an activator of Nrf2 signalling in Hct116 human colon carcinoma cells. An equivalent impact on non-tumour cells could be shown in total protein derived from CAPE treated FHC cells. The influence of CAPE on the Nrf2 pathway is known from literature. Incubation with CAPE increases the expression of the Nrf2 target gene heme oxygenase-1 in astrocytes [14] and induced Nrf2 translocation and ARE activation in rat renal epithelial cells [19]. *In vivo* CAPE could increase the measured levels of Nrf2 target genes expressed in the heart [15].

Next the effect of CAPE on DAF-16 activity was analysed using a DAF-16::GFP expressing transgenic *C. elegans* strain. CAPE treatment results in a significant increase in nematodes with a nuclear localisation of DAF-16 showing that the hydroxycinnamic acid derivative activates this transcription factor. This appeared to be of importance for the impact of CAPE on thermotolerance because in a SYTOX Green assay using a DAF-16 loss of function strain, no CAPE-mediated increase of thermotolerance was shown. A lifespan analysis using the same strain further revealed the dependence on DAF-16 for the life prolongation. The loss of functional DAF-16 even led to a decrease of lifespan after incubation with CAPE. This result could lead us to a possible mechanism. In the wild-type nematode, CAPE could induce a mild stress which would stimulate DAF-16 and thereby protection and a longer lifespan. In knock-out nematodes CAPE would only play the role of the stressor which would explain the shortened lifespan. The additional knock-down of SKN-1 using RNAi did not have any further influence. Thereby we were able to show that exposure of *C. elegans* to CAPE activates the transcription factor DAF-16 and that the induction of life prolongation by CAPE is dependent on the presence of this transcription factor. This shows a correlation between CAPE and insulin-like signalling in *C. elegans*.

Based on these results, we analysed the effect of the compound on FoxO4 (mammalian homologue of DAF-16) in Hct116 cells. This transcription factor is one of four known isoforms known, which are linked, e. g. to the regulation of cell cycle arrest, apoptosis, glucose metabolism and stress-resistance [35], [36]. Under cell culture or physiological conditions the cells are influenced by growth factors or insulin which lead to an AKT-mediated inhibitory phosphorylation of FoxO. Initially, we analysed the phosphorylation of FoxO4 at Ser193, a central phosphorylation site for AKT [37]. In case of an activation we would hence expect a decrease of phosphorylated FoxO4. However, incubation of Hct116 cells with CAPE (up to 40 µM) did not induce this effect, even so a slight tendency was perceived at the highest concentration. Next to the phosphorylation status, FoxO4 localisation gives information concerning activation [37]. Therefore we performed western blots of cytosolic and nuclear fractions. FoxO4 was not detected in the cytosol independent of the incubation with CAPE. Consistently unchanging low FoxO4 amounts were found in the nuclear fraction. In accordance with the unchanged FoxO4 phosphorylation status this shows that CAPE does not modulate FoxO4. Tough the possibility that another isoform is activated cannot be ruled out, it seems relatively unlikely because FoxOs are known to act in concert concerning defence against oxidative stress [35].

To the best of our knowledge we were able to show for the first time that CAPE increases lifespan of an organism: The hydroxycinnamic acid derivative prolongs lifespan by activation of the insulin-like DAF-16-signalling pathway in *C. elegans*, which is a different mechanism compared to the activation of the Nrf2-pathway already demonstrated in mammalian cells. Both pathways (SKN-1/Nrf2, DAF16/FoxO) are ageing-related redox-sensitive pathways [38], but it has to be further evaluated, why CAPE does activate DAF-16 instead of SKN-1 in the nematode. The insulin-like pathway is very well conserved across species as is the SKN-1 pathway, but there are also differences. Studies have identified three distinctions between regulation and function of SKN-1 in *C. elegans* and Nrf2 in mammals: i) DNA binding domains, ii) mechanisms of transcriptional activation, and iii) regulation of protein levels [39]. Furthermore, a cross-talk between the insulin-like pathway and the SKN-1 pathway has been reported in *C. elegans* (overview in [40]) which has not been reported for mammalian cells. Another relevant difference between the species might be the absence of a *C. elegans* homologue to human NFκB (reviewed by [41]) which is known to be inhibited by CAPE in mammalian cells [11]. Future deeper insights into cross-talk of signal transduction might shed a different light onto our results. Regarding the molecular mechanism by which CAPE modulates insulin-like signalling pathway in C. elegans, a modulation of the protein kinase Akt [42]–[44] is suggested. At present modulation of the insulin-like signalling pathway was found to be a pivotal effect of CAPE in *C. elegans*, this has to be taken into account for the investigation of the molecular mechanisms of the traditional use of propolis.

## Supporting Information

Figure S1 CAPE-mediated reduction of ROS accumulation in wild type C. elegans: Determination of the concentration-dependence. The antioxidative effect was measured using an in vivo DCF assay: Nematodes were incubated with different concentrations of CAPE (25, 50 and 100 µM) for 2 days and were then subjected to thermal stress (37°C); the DCF fluorescence intensity correlates with the intracellular ROS concentration; data are the mean ± SD, n = 3 with 16 individuals per group and experiment, *: p<0.05 and **: p<0.01 versus corresponding DMSO-treated group (0 µM).
(TIFF)
