# Supplementary figures and images for: Caffeic Acid Phenethylester Increases Stress Resistance and Enhances Lifespan in Caenorhabditis elegans by Modulation of the Insulin-Like DAF-16 Signalling Pathway
Source: PLoS One. 2014 Jun 25;9(6):e100256. doi: 10.1371/journal.pone.0100256 (PMC4070918; doi:10.1371/journal.pone.0100256)

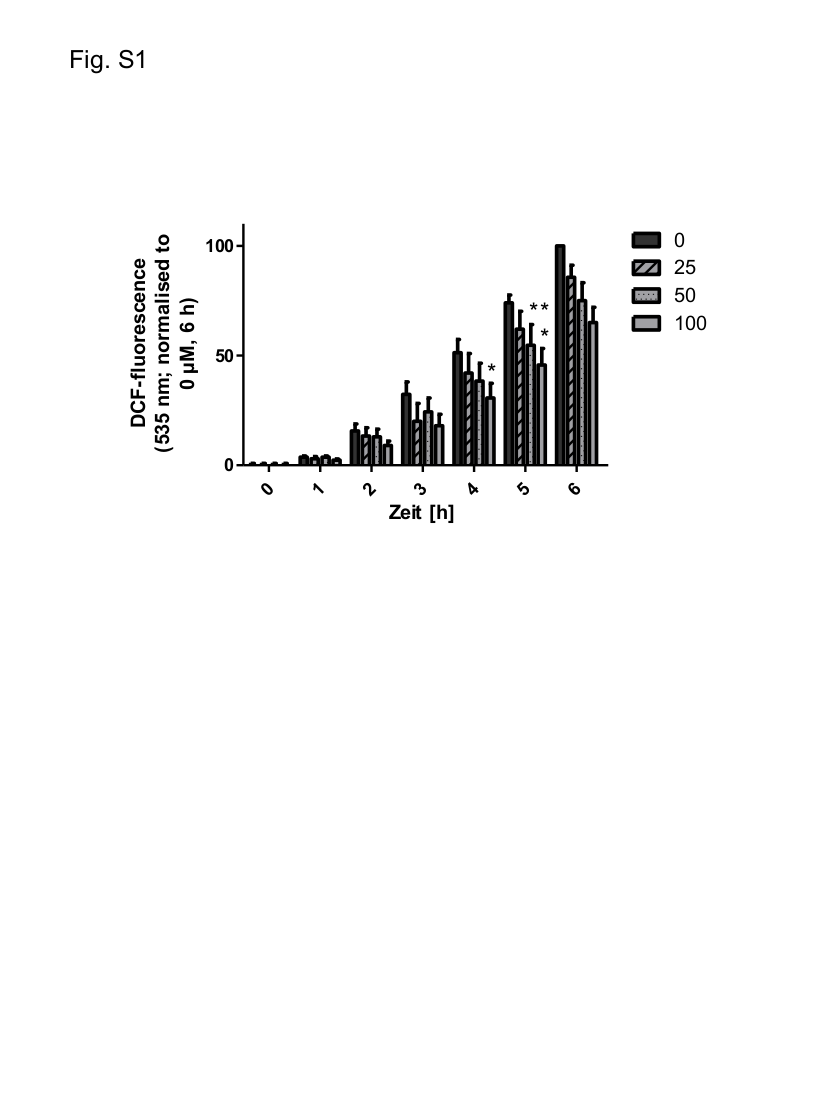

Supplement: Figure S1 — CAPE-mediated reduction of ROS accumulation in wild type C. elegans: Determination of the concentration-dependence. The antioxidative effect was measured using an in vivo DCF assay: Nematodes were incubated with different concentrations of CAPE (25, 50 and 100 µM) for 2 days and were then subjected to thermal stress (37°C); the DCF fluorescence intensity correlates with the intracellular ROS concentration; data are the mean ± SD, n = 3 with 16 individuals per group and experiment, *: p<0.05 and **: p<0.01 versus corresponding DMSO-treated group (0 µM). (TIFF) [file pone.0100256.s001.tiff]
